# In Vivo Base Editing for Neonatal Inborn Errors of Metabolism: Clinical Progress, N-of-1 Therapy, and the Ethics of Bespoke Genetic Medicine

**DOI:** 10.7759/cureus.114354

**Published:** 2026-08-11

**Authors:** Oscar Mingtian Li, Yijiang Li, Summer Xia tian Li

**Affiliations:** 1 Biomedical Engineering, Zweistein Innovation Lab, Active Care &amp; Education in Sports and Outreach Foundation (ACESO Foundation), Dublin, USA; 2 Cardiovascular Medicine, Zweistein Innovation Lab, Active Care &amp; Education in Sports and Outreach Foundation (ACESO Foundation), Dublin, USA; 3 Human Biology, Zweistein Innovation Lab, Active Care &amp; Education in Sports and Outreach Foundation (ACESO Foundation), Dublin, USA

**Keywords:** base editing, cps1 deficiency, gene editing, genetic therapy, inborn errors of metabolism, lipid nanoparticles, n-of-1 therapy, urea cycle disorders

## Abstract

Severe neonatal-onset inborn errors of metabolism (IEMs), such as urea cycle disorders including carbamoyl phosphate synthetase 1 (CPS1) deficiency and the classic organic acidemias, present within days of birth with metabolic decompensation that carries high early mortality and, in survivors, a substantial burden of neurologic injury despite optimal medical management. Because most cases arise from defined point mutations, these disorders are conceptually well suited to one-time genetic correction. Base editing, which installs precise single-base changes without generating double-strand DNA breaks, and its companion technology, prime editing, have moved rapidly from laboratory description to in vivo demonstration in animals and, most recently, to a single human patient. In 2025, an infant with CPS1 deficiency ('KJ') received a bespoke lipid nanoparticle-delivered base-editing therapy designed and manufactured for that individual's specific variant, becoming the first reported recipient of a customized in vivo gene-editing medicine.

This review synthesizes the clinical rationale for genetic correction of neonatal IEMs, the mechanistic basis and delivery strategies (lipid nanoparticles and adeno-associated virus) that make in vivo base editing feasible, and the preclinical evidence that preceded the first human case. We then examine the ethical dimensions of bespoke 'N-of-1' genetic medicine: the somatic versus germline distinction; consent for a non-autonomous neonate; equity, cost, and scalability; and the evolving regulatory pathway for individualized therapies. We conclude by distinguishing what has been proven in a single patient and in preclinical models from what remains speculative and by outlining what would need to generalize for a single case to become a platform.

## Introduction and background

Inborn errors of metabolism (IEMs) are individually rare but collectively important monogenic disorders in which a defective enzyme or transporter disrupts a metabolic pathway. The most severe forms declare themselves in the neonatal period, when the newborn, no longer buffered by placental clearance, accumulates toxic metabolites within hours to days of birth. Urea cycle disorders (UCDs), which impair the hepatic detoxification of ammonia, and the classic organic acidemias are archetypal examples: affected neonates present with encephalopathy, feeding intolerance, and rapidly progressive metabolic crisis that can be fatal or leave survivors with permanent neurologic injury [1,2].

Carbamoyl phosphate synthetase 1 (CPS1) deficiency, a proximal and among the most severe UCDs, illustrates the therapeutic gap. Even with prompt recognition and intensive management, protein restriction, nitrogen-scavenger drugs, hemodialysis for acute hyperammonemia, and eventually liver transplantation, neonatal-onset disease carries an estimated 50% mortality in early infancy, and the standard of care is lifelong, burdensome, and only partially protective [1,3]. Because such disorders typically result from specific, identifiable point mutations in a single gene, they are conceptually ideal candidates for a durable, one-time genetic correction that restores the missing enzymatic function rather than managing its consequences.

The tools to attempt such a correction have matured rapidly. Base editing enables the precise conversion of one DNA base pair to another without introducing the double-strand breaks that limit the safety of first-generation nuclease editing, and it can be delivered to the liver in vivo using lipid nanoparticles (LNPs) or viral vectors [4-6]. In 2025, these advances converged in the first report of a bespoke in vivo base-editing therapy designed, manufactured, and administered for a single infant with CPS1 deficiency [3]. For readers new to the field, the practical difference between the two leading precision tools is straightforward: a base editor chemically converts one DNA letter into another within a small target window and is therefore best suited to correcting single-nucleotide substitutions, whereas a prime editor uses a short RNA template to write in a specified new sequence and can additionally make small insertions and deletions [7-9]. The liver is a particularly favorable target for both approaches. It receives a large share of cardiac output and takes up systemically infused lipid nanoparticles efficiently, and the enzymes deficient in urea cycle disorders and the classic organic acidemias are normally expressed in hepatocytes, so that correcting a fraction of those cells can restore enough metabolic flux to alter the clinical phenotype [9-11]. This review surveys the clinical problem, the enabling technology and delivery systems, the preclinical and single-patient evidence, and the ethical and regulatory questions raised by individualized 'N-of-1' genetic medicine. Throughout, we distinguish carefully between what has been demonstrated and what remains aspirational.

## Review

Review methodology and scope

This article is a narrative review and is reported as such. No protocol was registered, no formal risk-of-bias instrument was applied, and full PRISMA 2020 reporting, which is designed for systematic reviews, was neither undertaken nor claimed [12]. The manuscript was instead prepared against the SANRA criteria for the quality of narrative review articles [13]. The search and selection process is stated explicitly below so that readers can judge the completeness of the evidence base for themselves.

Literature was identified by targeted searching of PubMed via the NCBI E-utilities interface on July 23, 2026; additional sources identified during peer review were incorporated and their bibliographic details verified against Crossref on August 4, 2026, which is the date of the final search. No other bibliographic database was searched, and no date limits were applied; the retrieved literature spans 2012 to 2025. Search terms combined three domains: the disease domain ("urea cycle disorder", "carbamoyl phosphate synthetase 1 deficiency", "organic acidemia", "methylmalonic acidemia", "propionic acidemia", "phenylketonuria"); the technology and delivery domain ("base editing", "adenine base editor", "cytosine base editor", "prime editing", "lipid nanoparticle", "mRNA delivery", "adeno-associated virus", "in vivo genome editing"); and the ethics and regulatory domain ("N-of-1 therapy", "individualized therapy", "somatic versus germline editing", "drug regulation individualized therapies"). Reference lists of retrieved articles were screened by hand for additional primary sources.

Selection was purposive rather than protocol-driven, and the criteria that follow describe the principles applied rather than a prespecified protocol. We included peer-reviewed, English-language reports of (i) the development or engineering of base or prime editors; (ii) in vivo editing in mammalian models or in humans with a hepatic or metabolic target; (iii) delivery platforms relevant to hepatic editing; (iv) contemporary management guidelines or outcome cohorts for the metabolic diseases discussed; and (v) ethical, regulatory, or policy analysis of individualized genetic therapy. We excluded preprints, conference abstracts, non-English publications, editorials without substantive analysis, purely mechanistic in vitro work without a translational correlate, and studies of germline or embryo editing except where cited to delineate the somatic-germline boundary. Where several reports addressed the same finding, we cited the primary study and, where helpful for orientation, one authoritative review. Screening and selection were performed by a single reviewer without duplicate independent assessment, and every cited record was verified against its indexed metadata for a valid digital object identifier.

No quantitative synthesis was undertaken. Meta-analysis, meta-regression, and pooled effect estimation were not performed and would not be interpretable here: the evidence comprises heterogeneous preclinical studies with non-commensurable endpoints together with a single treated human patient, so pooled estimates, P values, and confidence intervals cannot be meaningfully derived [12]. Findings are therefore presented descriptively, and the resulting constraints on inference are set out in the Limitations section below.

The clinical problem: severe neonatal-onset inborn errors of metabolism

UCDs comprise defects in the six enzymes and two transporters of the hepatic urea cycle, the principal route for disposing of waste nitrogen as urea. Loss of function at any step causes hyperammonemia, which is directly neurotoxic. Consensus guidelines emphasize that neonatal-onset presentations are medical emergencies in which the duration and peak of hyperammonemia correlate with the severity of subsequent neurologic outcome, so that even survivors of the initial crisis frequently sustain cognitive impairment [1]. CPS1 deficiency, an autosomal-recessive defect in the first committed step of the cycle, is associated with particularly unfavorable outcomes, and cohort data document high rates of death and neurodevelopmental disability despite contemporary management [2].

The classic organic acidemias-methylmalonic acidemia (MMA) and propionic acidemia (PA)-arise from defects in the catabolism of branched-chain amino acids and other precursors, leading to accumulation of toxic organic acids, metabolic acidosis, and secondary hyperammonemia. Like UCDs, they present with neonatal crisis and are managed with protein-restricted diets, carnitine, and, in selected cases, cofactor supplementation, with published guidelines underscoring the difficulty of achieving durable metabolic control [14,15]. Across these conditions, medical therapy is chronic, exacting for families, and incompletely effective; liver transplantation can correct or attenuate the hepatic enzymatic defect but requires a suitable organ, carries perioperative and lifelong immunosuppression risks, and does not reverse pre-existing injury [1,10]. This combination of high morbidity, defined genetic cause, and a hepatocyte-centered pathophysiology motivates the search for a one-time corrective genetic therapy.

Base editing and prime editing: mechanism and rationale for point-mutation disease

Programmable genome editing derives from the demonstration that the bacterial Cas9 nuclease can be directed to an arbitrary DNA sequence by a short guide RNA [16]. Base editors were subsequently designed to make a defined chemical change to a single base while avoiding the double-strand break on which nuclease editing depends, so that the outcome no longer rests on which repair pathway the cell happens to use [17]. This distinction matters therapeutically, because the efficiency and predictability of templated repair, rather than the targeting step itself, has been the principal obstacle to correcting point mutations in vivo [18]. Base editors are engineered fusion proteins that couple a catalytically impaired CRISPR-Cas protein, which provides programmable DNA targeting but does not cut both strands, to a nucleotide-modifying enzyme. Cytosine base editors (CBEs), first described in 2016, use a cytidine deaminase to convert a targeted C·G base pair to T·A [4]. Adenine base editors (ABEs), reported in 2017, use a laboratory-evolved deoxyadenosine deaminase to convert A·T to G·C, thereby enabling correction of a large fraction of known pathogenic point mutations [5]. Because base editors do not rely on double-strand breaks or on a supplied DNA repair template, they largely avoid the insertions, deletions, and chromosomal rearrangements that can accompany nuclease editing, a property especially relevant when treating post-mitotic or slowly dividing tissues such as the liver [7].

Prime editing, described in 2019, extends the precision toolkit: a Cas9 nickase fused to a reverse transcriptase, guided by an extended prime-editing guide RNA, can write a broader range of edits-including all base-to-base conversions, small insertions, and small deletions-without double-strand breaks [8,19]. For the point mutations that underlie most severe IEMs, base editing is often the more mature and efficient option, whereas prime editing offers flexibility when the required change falls outside the scope of available base editors [7,8]. A recognized limitation of base editing is bystander editing, in which additional bases within the editing window are inadvertently changed; protein engineering has produced narrowed-window editors that reduce such collateral edits, improving the precision needed for therapeutic use [20]. The principal genome-editing modalities relevant to correcting point mutations are compared in Table 1.

**Table 1:** Genome-editing modalities relevant to correcting point mutations in metabolic disease Base editors and prime editors avoid double-strand breaks, reducing insertions, deletions, and rearrangements [7]; bystander editing within the editing window is a recognized limitation of base editing [20]. CRISPR: Clustered regularly interspaced short palindromic repeats; CBE: Cytosine base editor; ABE: Adenine base editor; PE: Prime editor; HDR: Homology-directed repair; NHEJ: Non-homologous end joining; pegRNA: Prime-editing guide RNA

Modality	Molecular mechanism	Double-strand break	Edit types possible	Reference no.
Programmable nuclease (CRISPR-Cas9)	Targeted double-strand break repaired by non-homologous end joining (NHEJ) (disruption) or homology-directed repair (HDR) (templated correction)	Yes	Insertions/deletions; templated substitutions	[7]
CBE	Catalytically impaired Cas fused to a cytidine deaminase; edits within a defined window	No	C·G → T·A	[4]
ABE	Impaired Cas fused to a laboratory-evolved adenosine deaminase	No	A·T → G·C	[5]
PE	Cas9 nickase fused to a reverse transcriptase; edit templated by an extended prime-editing guide (peg)RNA	No (single-strand nick)	All 12 substitutions; small insertions/deletions	[8,19]

In vivo delivery: lipid nanoparticle and viral platforms

**Figure 1:**
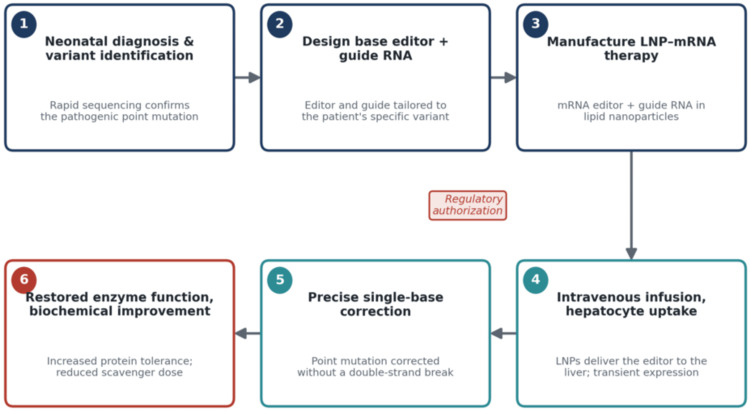
Workflow of a bespoke N-of-1 in vivo base-editing therapy for a neonatal inborn error of metabolism The patient-specific therapy is administered only after regulatory authorization. Steps proceed from neonatal diagnosis and variant identification, through design and LNP manufacture of the base editor, to intravenous infusion and hepatocyte uptake, precise single-base correction, and biochemical improvement (increased protein tolerance and reduced nitrogen-scavenger dose). LNP: Lipid-nanoparticle

Realizing base editing as a medicine requires delivering the editor to the correct tissue. Two platforms dominate in vivo delivery to the liver, the target organ for most metabolic IEMs. LNPs encapsulate messenger RNA (mRNA) encoding the base editor together with the guide RNA; after intravenous infusion, they are taken up preferentially by hepatocytes, where the editor is transiently expressed and then cleared [11]. This transient, non-integrating exposure is attractive for safety, because it limits the duration of editor activity and thereby the window for off-target editing, and it is the same class of technology validated at scale by mRNA-LNP vaccines [11]. AAV-delivered base editors have achieved editing across the liver and several other tissues in mice, establishing the reach of the platform [21]. Adeno-associated virus (AAV) vectors offer an alternative, providing durable episomal expression and well-characterized hepatic tropism, but they raise distinct concerns including pre-existing and treatment-induced immunity, vector-genome persistence, and constraints on cargo size that complicate packaging of large editor constructs [22,23]. The choice between transient LNP-mRNA delivery and vector-based expression involves a trade-off between control and durability that is central to the design of any in vivo editing therapy. Figure 1 outlines the end-to-end workflow of a bespoke in vivo base-editing therapy for a neonatal metabolic disease.

From preclinical proof-of-concept to the first human case

The path to human application was laid by successive in vivo demonstrations. A pivotal study showed that CRISPR base editors delivered by LNP could achieve near-complete, durable knockdown of PCSK9 in the liver of nonhuman primates after a single infusion, with stable reductions in circulating PCSK9 and low-density lipoprotein cholesterol (LDL-C) for at least eight months, establishing proof of concept for precise, single-nucleotide hepatic editing in a large animal [6]. An independent group reported concordant results with an adenine base editor delivered by LNP in macaques, with durable reductions in circulating PCSK9 and LDL-C after a single dose, providing replication across laboratories and editor chemistries [24]. Subsequent investigational work advanced PCSK9-directed base editing toward the clinic [25]. In a disease-correction paradigm, adenine base editing corrected the causative LMNA mutation in a mouse model of Hutchinson-Gilford progeria syndrome, reducing pathogenic progerin, rescuing vascular pathology, and substantially extending median lifespan after a single AAV-delivered dose-evidence that in vivo base editing can modify disease course, not merely a biomarker [26].

Correction of a hepatic metabolic defect had in fact been demonstrated earlier in mice with hereditary tyrosinemia type 1: a cytidine base editor delivered to adult animals corrected the pathogenic point mutation in the Fah gene and restored the metabolic phenotype [27], and adenine base editing subsequently achieved the same correction, with edited hepatocytes expanding selectively because the disease places uncorrected cells at a growth disadvantage [28]. Directly relevant to metabolic disease, several groups demonstrated in vivo correction of phenylketonuria in mice. Base editing delivered by mRNA-LNP corrected the common PAH variant and ameliorated the biochemical phenotype [29,30], and prime editing achieved efficient in vivo correction of the same high-prevalence variant [31]. Complementary work using patient-derived hepatocytes showed that gene correction of cells from inherited liver diseases is feasible and can restore function, reinforcing the hepatocyte as a tractable target [10,32]. These studies collectively established that precise, in vivo genetic correction of a metabolic defect in the liver was achievable in animal models. Representative in vivo base- and prime-editing studies that preceded the first patient-specific therapy are summarized in Table 2.

**Table 2:** Representative in vivo base- and prime-editing studies preceding the first patient-specific therapy A dash (—) indicates a delivery route not specified here. Outcomes are as reported in the cited studies and, for the human case, reflect a single patient with short follow-up. ABE: Adenine base editor; AAV: Adeno-associated virus; LDL-C: Low-density lipoprotein cholesterol; CRISPR: Clustered regularly interspaced short palindromic repeat; LNP: Lipid nanoparticle; PKU: Phenylketonuria

Authors and reference no.	Model	Target/disease	Editor	Delivery	Principal reported outcome
Musunuru et al., 2021 [6]	Nonhuman primate	PCSK9/hypercholesterolemia	ABE	LNP–mRNA	Durable PCSK9 and LDL-C reduction ≥8 months after a single infusion
Lee et al., 2022 [25]	Nonhuman primate and mouse	PCSK9	CRISPR base editor	LNP	Single-course base editing advanced toward clinical development
Koblan et al., 2021 [26]	Mouse (progeria)	LMNA/Hutchinson-Gilford progeria	Adenine base editor	AAV	Reduced progerin, rescued vascular pathology, extended lifespan
Brooks et al., 2023 [29]	Mouse	PAH/phenylketonuria (PKU)	Base editor	mRNA–LNP	Corrected the most frequent PKU variant; improved biochemistry
Yin et al., 2024 [30]	Mouse (Pah)	PAH/PKU	Base editor	—	Ameliorated metabolic and behavioral defects
Brooks et al., 2023 [31]	Mouse	PAH/PKU	Prime editor	—	Efficient in vivo correction of the frequent PKU variant
Musunuru et al., 2025 [3]	Human infant	CPS1/urea cycle disorder	Bespoke base editor	LNP–mRNA	First patient-specific in vivo editing; increased protein tolerance, halved scavenger dose, no serious adverse events (short follow-up)

Parallel clinical experience with nuclease editing established that CRISPR-based medicines could be administered to patients at all. Ex vivo editing of autologous hematopoietic stem cells produced durable, transfusion-independent responses in sickle cell disease and transfusion-dependent beta-thalassemia [33]. The first in vivo application, an LNP-delivered Cas9 targeting TTR in hereditary transthyretin amyloidosis, produced dose-dependent and sustained reductions in circulating transthyretin after a single infusion [34], and the same delivery platform was extended to KLKB1 in hereditary angioedema, where a single dose durably reduced attack frequency [35]. These programs are gene disruptions rather than corrections, but they validated the systemic delivery of an editor to human hepatocytes, which is the step on which a bespoke corrective therapy depends.

**Figure 2:**
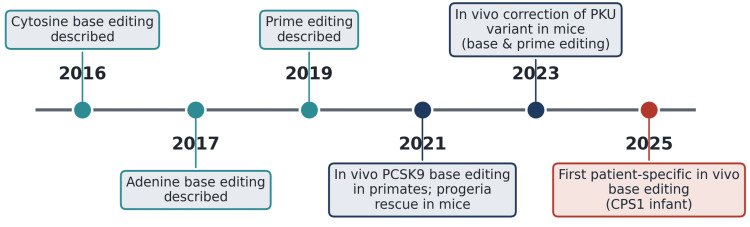
Chronology of key milestones in the development of in vivo base and prime editing toward treatment of neonatal metabolic disease The years denote the studies cited in the text: cytosine base editing (2016) [4] and adenine base editing (2017) [5]; prime editing (2019) [19]; in vivo PCSK9 base editing in primates [6] and progeria rescue in mice [26] (2021); in vivo correction of a phenylketonuria variant in mice by base editing [29] and prime editing [31] (2023); and the first patient-specific in vivo base-editing therapy in an infant with CPS1 deficiency (2025) [3]

This groundwork enabled the landmark single-patient case reported in 2025. After a neonate received a diagnosis of severe CPS1 deficiency, a disease with an estimated 50% mortality in early infancy, investigators developed a customized LNP-delivered base-editing therapy targeting that patient's specific variant. Following regulatory authorization, the infant ('KJ') received two infusions at approximately seven and eight months of age. In the seven weeks after the first infusion, the patient tolerated an increased amount of dietary protein and a reduction of the nitrogen-scavenger medication to half the starting dose, without unacceptable adverse events and despite intercurrent viral illnesses; no serious adverse events were reported [3]. The authors framed the result cautiously, noting that longer follow-up is warranted to assess safety and efficacy [3]. This case represents the first administration of a bespoke in vivo gene-editing medicine to an individual patient and a proof of principle that a therapy can be conceived, engineered, and delivered on a timescale relevant to a rapidly progressive neonatal disease while remaining, importantly, a single-patient experience rather than evidence of generalizable efficacy [3,36]. The chronology of key milestones leading to this case is summarized in Figure 2.

Off-target editing and other safety considerations

The safety case for base editing rests on more than the absence of double-strand breaks, and three classes of unintended edits must be assessed for any candidate therapy. Bystander editing, noted above, alters additional bases of the same type within the editing window and is addressed by narrowed-window editor variants [20]. Cas-independent deamination occurs when the deaminase domain acts on transiently single-stranded DNA elsewhere in the genome; this activity was quantified in mouse embryos, where a CBE produced a substantial excess of genome-wide single-nucleotide variants [37]. Both CBE and ABE can also deaminate cellular RNA, generating transcriptome-wide off-target edits that are transient but measurable [38]. Both liabilities have proved tractable to protein engineering, since deaminase variants with reduced intrinsic affinity for single-stranded nucleic acid substantially lower Cas-independent editing while preserving on-target activity [39]. Transient mRNA-LNP delivery mitigates all three classes by limiting the duration of editor exposure, which is one reason it has been favored over vector-driven constitutive expression in hepatic applications [9,11]. Beyond editing fidelity, the practical safety agenda for a neonatal recipient includes the innate immune response to the nanoparticle and its cargo, the durability of correction in a growing liver whose hepatocytes continue to divide, and the possibility that a small edited fraction becomes insufficient as body mass increases; none of these can be resolved by a single treated patient. These technical questions are necessary but not sufficient. Because such therapies are built for one child at a time, under time pressure, and outside the population-based framework in which medicines are normally evaluated, they raise ethical and regulatory questions that are as determinative of the field's trajectory as the editing chemistry itself.

Ethics of bespoke genetic medicine

Individualized genetic therapies raise ethical questions that differ in degree, and sometimes in kind, from those of conventional drug development. A foundational distinction is between somatic and germline editing. The therapies discussed here act on somatic cells, principally hepatocytes, and are not intended to be heritable; this places them within a broad ethical consensus that supports somatic genome editing to treat serious disease while continuing to regard heritable germline modification as ethically fraught and, in many jurisdictions, prohibited [40]. A position statement developed by the American Society of Human Genetics with international collaborators reached a compatible conclusion, holding that it is at present inappropriate to perform germline editing that culminates in a human pregnancy, while finding no reason to prohibit in vitro germline research under appropriate oversight and setting out the conditions of evidence, medical rationale, and transparent public process that any future clinical application would have to satisfy [41]. Maintaining this boundary, including attention to whether any editing reaches germ cells, is essential to the legitimacy of the field.

A second challenge is consent. A critically ill neonate cannot provide assent, and parents must weigh a novel, largely untested intervention against a disease of high mortality, often under acute time pressure. The rationale for proceeding is strongest precisely when the prognosis is grave, and alternatives are limited, but this same urgency constrains deliberation and heightens the risk of therapeutic misconception. The earlier 'milasen' experience, a customized antisense oligonucleotide designed for a single child with a rare neurodegenerative disease, illustrated both the promise and the ethical complexity of individualized therapy, including uncertainty about efficacy, the weight of parental hope, and the difficulty of interpreting outcomes in a single patient [42]. Transparent communication that a bespoke therapy is investigational, that benefit is unproven, and that risks are incompletely characterized is a central obligation.

Finally, equity, cost, and scalability are pressing. A therapy engineered for one patient's variant entails substantial expense and specialized infrastructure, raising the concern that bespoke genetic medicine could become available only to a fortunate few or at centers of concentrated expertise. Whether the underlying platform can be standardized, so that the reagents change while the manufacturing and safety framework stay fixed, will largely determine whether these therapies remain singular events or become broadly accessible [36,43]. Absent such standardization, the prospect of durable one-time cures for some children could paradoxically widen disparities in care.

Regulatory pathway for N-of-1 therapies

Conventional drug regulation presupposes a fixed product studied in populations through sequential clinical trials. A therapy designed for a single patient does not fit this model: there is no population in which to establish efficacy, and the product itself is unique. Regulators and investigators have begun to articulate frameworks for such 'N-of-1' interventions, emphasizing that oversight should scale with risk and that a platform-based approach may be the most workable path-regulating the shared, validated manufacturing and delivery process while allowing the patient-specific component to vary, rather than treating every individualized therapy as an entirely new product [43]. This reasoning, advanced in the context of individualized antisense oligonucleotides, extends naturally to bespoke base-editing therapies [42,43].

The CPS1 case operationalized elements of this thinking: the therapy was developed and manufactured for one patient and administered only after regulatory authorization specific to that individual [3]. Realizing a scalable future will require prospective agreement on what evidence is sufficient before first-in-human use of a bespoke editor, how to define and validate the reusable platform, how to monitor for off-target and long-term effects in populations too small for conventional trials, and how to share safety data across individual cases so that each patient's experience informs the next. A platform-oriented regulatory model, coupled with rigorous long-term pharmacovigilance, is the most plausible route from isolated compassionate-use cases to a generalizable therapeutic class [36,43]. European work on individualized antisense oligonucleotides has since translated this reasoning into concrete procedural proposals, covering the selection of eligible patients, the minimum preclinical evidence required before first administration, the conduct of informed consent under profound uncertainty, and mechanisms for pooling outcome data across separately treated individuals; these proposals map closely onto the needs of bespoke editing programs [44].

Limitations

Several limitations qualify this review. First, it is narrative rather than systematic: a single bibliographic database was searched, selection was purposive and carried out by one reviewer without duplicate independent screening, and no protocol was registered, so selection bias toward prominent journals and highly cited work cannot be excluded [13]. Second, no formal risk-of-bias appraisal was applied to the included studies and no quantitative synthesis was possible, so the evidence is summarized descriptively rather than graded [12]. Third, the clinical evidence base is a single patient with approximately seven weeks of reported follow-up; nothing in this literature supports inference about efficacy, durability, or the incidence of rare adverse events at a population level [3]. Fourth, most efficacy data derive from rodent and nonhuman primate studies, and interspecies differences in hepatocyte turnover, immune response, and nanoparticle biodistribution limit their predictive value for a human neonate. Fifth, the field is moving quickly, and work appearing after the final search date, including any further treated patients, is necessarily absent. Finally, the ethical and regulatory discussion reflects frameworks in high-income regulatory environments and may not transfer to jurisdictions with different oversight structures or resource constraints.

## Conclusions

Severe neonatal-onset IEMs remain among the most lethal and disabling monogenic diseases, and their defined point-mutation etiology makes them compelling targets for precise genetic correction. Base editing, with prime editing as a complementary tool, provides a mechanism to install the necessary single-base changes without double-strand breaks, and LNP and AAV delivery make in vivo hepatic correction feasible. On that preclinical foundation, the 2025 CPS1 case established for the first time that a bespoke in vivo base-editing therapy can be designed and safely administered to an individual infant.

These achievements must be read with precision about their limits. The human evidence is a single patient with short follow-up; the authors themselves emphasize that longer observation is required to judge safety and efficacy, and no claim of cure is warranted. What generalizes from this experience is not a finished therapy but a template: a reusable platform of editing chemistry, delivery, manufacturing, and regulatory process into which a patient-specific correction can be inserted. Whether that template can be made safe, equitable, and affordable at scale and validated through frameworks suited to individualized medicine will determine whether in vivo base editing becomes a broadly available option for neonatal metabolic disease or remains a remarkable but singular achievement. Continued, transparent reporting of each treated patient, within a shared safety framework, is the responsible path forward.
